# Impact of quorum sensing signaling molecules in gram-negative bacteria on host cells: current understanding and future perspectives

**DOI:** 10.1080/19490976.2022.2039048

**Published:** 2022-02-19

**Authors:** Yingping Xiao, Huicong Zou, Jingjing Li, Tongxing Song, Wentao Lv, Wen Wang, Zhenyu Wang, Shiyu Tao

**Affiliations:** a State Key Laboratory for Managing Biotic and Chemical Threats to the Quality and Safety of Agro-products and Institute of Agro-product Safety and Nutrition, Zhejiang Academy of Agricultural Sciences, Hangzhou, China; b College of Animal Sciences and Technology, Huazhong Agricultural University, Wuhan, China; c State Key Laboratory of Animal Nutrition, College of Animal Science and Technology, China Agricultural University, Beijing, China

**Keywords:** Quorum sensing, gram-negative bacteria, signaling molecule, host cell, gut health

## Abstract

Quorum sensing is a molecular signaling-based communication mechanism in prokaryotes. In the basic mode, signaling molecules released by certain bacteria are sensed by intracellular receptors or membrane-bound receptors of other members in the community, leading to the collective isogenic signaling molecule synthesis and synchronized activities. This regulation is important for the symbiosis of the bacterium with the host, as well as virulence and biofilm formation. Notably, quorum sensing signaling molecules are not only able to control microbial community behavior but can likewise regulate the physiological status of host cells. Here, we provide a comprehensive review of the importance of quorum sensing signaling molecules in gram-negative bacteria in regulating host cell function and gut health, and suggest possible opportunities for application in combating human and animal diseases by blocking the pathways through which quorum sensing signaling molecules exert their functions.

## Graphical abstract

**Figure:**
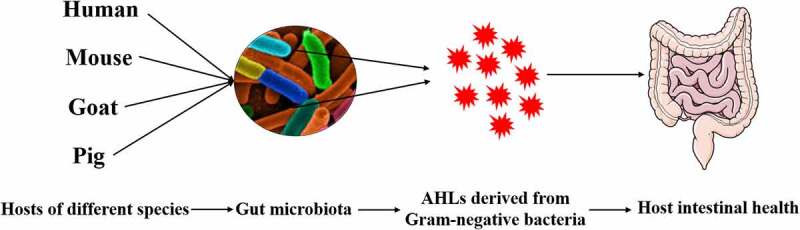

## Introduction

The international academic community calls a system in which multiple microorganisms live together a microbial community, also known as the microflora. However, many years ago, researchers discovered that bacteria communicate with each other, and through communication, they gain the ability to coordinate their operations.^1^^, 2^ Bacterial communication mainly relies on microbial quorum sensing (QS). The concept of QS, first proposed by Fuqua et al., refers to the sensing phenomenon that can only occur when the number of bacteria reaches a certain density.^3^ It was reported that QS is present in both gram-negative and gram-positive bacteria, and elevated local concentrations of QS signaling molecules (QSSMs) are often observed in bacterial infections.^4^ QS has now become a major research area in the field of microbiology. In the 1960s, Tomasz et al. discovered the first QSSM in *Streptococcus pneumoniae*.^5^ A subsequent study has shown that the QS system is widely present in various microbial populations.^6,7^ With new research developments, investigators have found that QSSM can not only regulate the group behavior of microorganisms, but also regulate the expression of virulence factors in human pathogenic bacteria, which has gradually attracted attention in the field of public health.

Bacterial QS, also known as self-induction, is a mechanism by which bacteria exchange intracellular or intercellular information, coordinate population behavior, and regulate gene expression, all of which depend on population density.^8^ When a bacterial community reaches a certain density, it turns on the expression of genes related to bacterial community density by secreting diffusible signaling small molecules (also known as QSSMs). QSSMs diffuse into the environment, and when the signaling molecules in the environment reach a certain threshold concentration, they induce the expression of specific genes in bacteria that are dependent on cell density, thus causing bacteria to exhibit new behavioral characteristics on a community scale such as biological luminescence, regulation of virulence factor secretion, budding spore formation or biofilm formation, cell differentiation, motility, and extracellular polysaccharide formation.^9,10^ A variety of microbial-related QSSMs have been identified. For gram-negative bacteria, most QSSMs belong to the N-acyl-homoserine lactone (AHL) family. The main differences among different AHLs are the length of the N-side chain, the substituents at the 3-carbon position, and the presence or absence of one or more unsaturated bonds in the side chain.^11,12^

Previous studies on QS have mostly focused on the interactions between microorganisms. In recent years, the direct effects of QSSMs on host cell functions have also attracted widespread attention.^13–15^ QSSMs are lipid-soluble small molecules that can easily penetrate into cells to affect cellular functions.^16^ Here, we summarize the current knowledge on microbial QS from national and international studies. Based on recent advances in the understanding of QS, we also highlight the importance of the reciprocal effects between QSSM AHL and host cells, including the effects and potential mechanisms of AHL in mammalian host cells, the relationship between AHL and intestinal health, and the mechanism of intestinal barrier dysfunction by AHL. Multiple targets of microbial QSSM may provide new possibilities for the diagnosis and treatment of bacterial infectious diseases.

## Characteristics of gram-negative bacterial QSSM

i) Small molecular weight: Gram-negative bacterial QSSM, such as AHL and its derivatives, oligopeptides, and γ-butyrolactones, are all small molecules that can freely enter and exit the cell or be secreted into the environment by oligopeptide permease and accumulate in the environment. ii) Species specificity: AHL in gram-negative bacteria has extraordinary specificity. Typically, gram-negative bacteria use LuxR-type receptors, a cytoplasmic transcription factor that detects AHLs produced by the chaperone LuxI-type synthases. LuxR proteins use amino acid variation and flexibility in the binding pocket to achieve AHL binding specificity.^7^ iii) Dependence on growth period and bacterial density: Generally, gram-negative bacterial QSSM accumulation in the environment reaches a higher concentration during the logarithmic or stable phase of bacterial growth, when it regulates the expression of the most genes. Moreover, the supernatant from a stable phase of bacterial growth can induce changes in the physiological state of bacteria during the incubation phase (lower bacterial density). iv) Regulatory role in gram-negative bacterial infection: Many gram-negative bacterial QSSM-producing bacteria belong to the family of plant and animal pathogenic or symbiotic bacteria, which play important regulatory roles in the interactions between bacteria and hosts.

## Gram-negative bacterial QS system

Gram-negative bacteria can release a class of small molecule signaling factors (AHL) into the surrounding environment that can move freely in and out of bacteria, and AHL plays a key role in the QS system.^7,17^ An informative table of different gram-negative bacterial QSSMs and function was shown in Table 1.

**Table 1.** Informative table of different gram-negative bacterial QSSMs and function

Bacterium	QSSMs	Function	References
*Vibrio fischeri*	C8-HSL, 3-oxo-C6-HSL	Bioluminescence	^18^
*Vibrio harveyi*	3-OH-C4-HSL	Protease production	^19^
*Pseudomonas aeruginosa*	C4-HSL, 3-oxo-C12-HSL	Rhamnolipid, protease, and virulence factor production	^20–22^
*Vibrio vulnificus*	C4-HSL	Antibiotic resistance	^23,24^
*Aeromonas salmonicida*	C4-HSL, C6-HSL	Antibiotic resistance, virulence factor production	^25,26^
*Yersinia ruckeri*	C8-HSL, 3-oxo-C8-HSL	Virulence factor production	^25,27^
*Agrobacteriium tumefaciens*	3-oxo-C8-HSL	Conjugational transfer	^28^
*Vibrio anguillarum*	3-oxo-C12-HSL	Protease production	^29^

**Figure 1.**
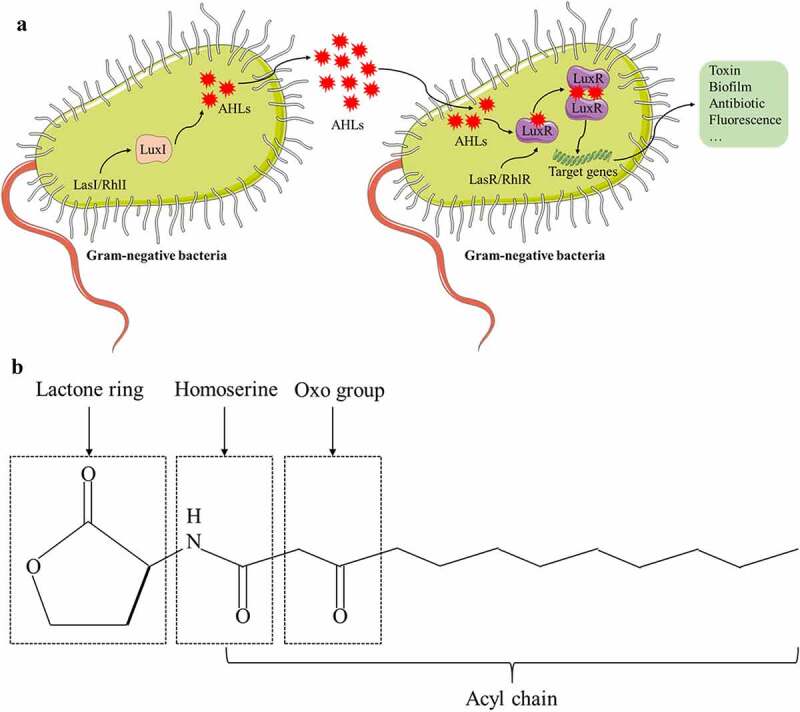
Quorum sensing signaling in gram-negative bacteria. (A) N-acyl-homoserine lactones (AHLs) are QSSM that communicate with each other in gram-negative bacteria. LasI/RhlI directs LuxI to synthesize AHLs, which diffuse freely outside the bacterial membrane and bind to their receptor LuxR when AHLs accumulate to a threshold concentration. The LuxR-AHLs complex activates the expression of target genes, triggering the occurrence of toxins, biofilms, antibiotics, or fluorescence. (B) Chemical structure of a typical AHL: N-3-oxododecanoyl-homoserine lactone (3-oxo-C12-HSL).

AHL was first discovered in the bioluminescence system of the marine bacterium *Vibrio fischeri*, and this phenomenon is positively correlated with the population density of the bacteria.^18^ For gram-negative bacteria, the QS system consisting of LuxI/LuxR is the most extensively studied. Taking *Vibrio fischeri* as an example, the LuxI homologous gene of the bacterium is responsible for the synthesis of QSSM AHL, whereas the LuxR homologue is activated as the AHL receptor to regulate the transcription of diverse downstream genes.^30,31^ Inter-microbial signaling mediated by AHL can regulate many functions of gram-negative bacteria such as the production of virulence factors, biosynthesis of antibiotics and extracellular polysaccharides, cell clustering, and entry into the stable growth phase. During the exponential growth of bacteria, AHL is synthesized in the cytoplasm and diffuses through the cytomembrane, thereby reaching the outside of bacteria and accumulating in the environment. When AHL reaches a specific critical concentration, LuxR binds to AHL to form the LuxR-AHL complex, and this complex can activate the promoter of related genes and eventually initiate the transcription of target genes ^32^ (Figure 1). LuxR stabilizes its structure by using AHL as a folding switch and degrades automatically when AHL is absent.^33^ Many bacteria with QS can produce more than one type of AHL molecule. For example, *Pseudomonas aeruginosa* can produce both C4-HSL and 3-oxo-C12-HSL, and the functions and mechanisms of different types of AHL molecules can vary in their regulation of the population behavior of microorganisms.^34^ Furthermore, several investigators have discovered that some bacteria lack genes that have functions similar to that of LuxI, rendering the bacteria unable to synthesize AHL by themselves. Interestingly, these bacteria encode LuxR<apos;>s homologous protein, SdiA,^35^ which can sense various population signals produced by other bacteria and regulate the expression of its own QS-related genes through an “eavesdropping” mechanism.^36^

In the QS system of gram-negative bacteria with AHL as the QSSM, the signal transduction pathway is diverse. Presently, *Pseudomonas aeruginosa* is the most mature, and it mainly contains four sets of QS systems. The first set is the lasR/lasI system, consisting of the transcriptional activator lasR and the acetylhomoserine lactone synthase lasI protein.^37^ LasI directs the synthesis of QSSM N-3-oxododecanoyl-homoserine lactone (3-oxo-C12-HSL) and secretes it into the extracellular compartment during active transport. It can bind lasR at a certain threshold concentration and activate gene transcription of virulence factors, including alkaline protease, exotoxin A, and elastase, which can increase the expression of virulence genes in *Pseudomonas aeruginosa*. The second set is the rhlR/rhlI system.^38^ RhlR is a transcriptional regulator. RhlI encodes AHL synthetase. The system produces homoserine lactone QSSM with a structure of C4-HSL, which can freely pass through the cell membrane and regulate the expression of a large number of genes, such as chitinase, cyanide, pyocyanin, and other genes. The Pseusomonas quinolone signal (PQS) is the third QS system for *Pseudomonas aeruginosa* recently discovered.^39,40^ The signal molecule of the quinolone signal system has antibacterial activity and is insoluble in water. The mechanism by which it uses inter-bacterial signal transduction is still unclear. It may involve the transmission of PQS signals between bacteria through an “exocytosis”-like transport mechanism. PQS can connect the two systems of lasR/lasI and rhlR/rhlI. On the one hand, lasR/lasI and rhlR/rhlI control the generation of PQS. On the other hand, PQS affects the gene expression of lasR/lasI and rhlR/rhlI. There is a delicate balance between the two. Furthermore, PQS plays a role in regulating bacterial density and releasing virulence factors. In addition to the above three QS systems, another *Pseudomonas aeruginosa* QS helper system, the GacS/GacA system, has been recently discovered and reported to play important roles in improving the ability of bacteria to travel, releasing theobromine sodium acetate, and promoting biofilm formation.^41^

## Effect of QSSM AHL on host cells and potential mechanisms

### Impact of AHL on cell apoptosis

QSSMs are a class of small molecules that are lipid soluble. They can reach mucosal epithelia or subcutaneous tissues by diffusion, even diffusing into the vasculature.^42^ It has been shown that AHL, the QSSM of gram-negative bacteria, can affect the function of host eukaryotic cells. Due to their lipid solubility, AHL can rapidly enter mammalian cells and induce apoptosis. As such, AHL plays a direct role in inducing apoptosis in host cells. AHLs can quickly initiate apoptosis of mouse embryonic fibroblasts, activate caspases-3/7 and 8, depolarize the mitochondrial membrane potential, and release cytochrome c from mitochondria into the cytoplasm, leading to cell and nuclear shrinkage, and ultimately cell death.^43^ Exposure of the respiratory epithelium to AHLs for only 1 hour results in the disintegration of tight junctions between epithelial cells. However, a membrane-penetrating pan-caspase inhibitor (Z-VAD-FMK) has been reported to block this damage, suggesting that the breakdown of tight junctions is an early event in apoptosis that is initiated by AHL.^44^ Low concentrations of AHL were sufficient to reduce the viability of undifferentiated Caco-2 cells and induce apoptosis by inhibiting AKT phosphorylation, whereas AKT overexpression partially reversed apoptosis.^45^ Furthermore, mucus protein mucin 3 (MUC3) protects against damage caused by AHL in epithelial cells.^46^ The pro-apoptotic effect of AHL is related to intracellular Ca^2+^ signaling. The pretreatment of cells with thapsigargin, an inhibitor of Ca^2+^ uptake into the endoplasmic reticulum, for 10 min can protect the barrier from damage by AHL and reduce apoptosis.^44^ AHL strongly induced apoptosis of macrophages and neutrophils.^47–50^ Furthermore, AHL promoted sperm apoptosis in a dose-dependent manner, thus reducing their motility.^51^ AHL also has a strong pro-apoptotic effect on cancer cells.^52^

### Impact of AHL on immunity

Depending on the acyl chain length, double bonds, and concentration, AHL can affect the host innate immune system to varying degrees. For macrophages, in general, AHL decreases their inflammatory response in a diverse manner, leading to chronic infection with *Pseudomonas aeruginosa*. It has been shown that *Pseudomonas aeruginosa*-produced AHL exerted an anti-inflammatory response in RAW264.7 mouse macrophages in a dose-dependent manner. At the same time, AHL induced a decrease in the concentration of TNF-α and an increase in the concentration of IL-10 secreted by RAW264.7 cells.^53^ In addition, it has been reported that macrophages have stronger phagocytic activity in the presence of AHL.^54^ For dendritic cells (DCs), the effects are different and depend on the cell type. In the presence of AHL, stimulation with lipopolysaccharides inhibited the secretion of pro-inflammatory cytokines IL-12 and interferon-γ in both human and mouse DCs. However, the same concentrations of AHL and lipopolysaccharides only enhanced the ability of human DCs, but not mouse DCs, to secrete IL-10.^55–57^ AHL also has a certain degree of influence on host adaptive immunity. AHL can inhibit mitogen-stimulated ^58^ and antigen-stimulated ^59^ T lymphocyte proliferation and function, as well as regulate antibody production by B lymphocytes.^60^ Furthermore, AHL can induce apoptosis in Jurkat cell lines via the mitochondrial pathway,^61^ thus inhibiting the activation and proliferation of DCs and T cells and downregulating the expression of costimulatory molecules on DCs.^62,63^

### Cell-type specific and dose-specific by which AHL affects host cell function

The induction of apoptosis in mammalian cells by AHL is distinctly cell specific. For example, numerous in vitro studies have shown that AHL can induce apoptosis in tracheal epithelial cells, breast cancer cells, macrophages, and neutrophils, but not in liver Hep2 and lung CCL185 epithelial cell lines.^64–68^ The regulation of immunity in mammalian cells by AHL is also cell type specific, with some studies reporting that AHLs can upregulate inflammatory cytokines,^69–71^ whereas other studies demonstrating that AHLs can reduce inflammation in cells.^60,72–75^ In addition, the effects of AHL on host cell function are similarly dose-specific. At higher concentrations (> 25 μM), intracellular events may predominate, but at lower concentrations (< 10 μM), more sensitive receptor-driven effects will predominate.^76^ Relatively low concentrations (between 10 to 30 μM) of AHL can reduce cell viability by inhibiting AKT phosphorylation in undifferentiated Caco-2 cells accompanied by apoptosis.^77^ The gastrointestinal tract is considered the most important reservoir of AHL-producing *Pseudomonas aeruginosa* in clinical settings, which is associated with higher mortality rates of patients. Human clinical study has demonstrated the ability of *Pseudomonas aeruginosa* to adhere to and penetrate intestinal epithelial cells as well as to form biofilms.^78^ The AHL is present in *Pseudomonas aeruginosa* biofilms in concentrations as high as 300–600 µM.^79^ Thus, the concentrations used in the studies of AHL affecting host cell function that we listed previously are of physiological significance, and these studies have contributed to guiding clinical practice.

### Potential mechanisms by which AHL affects host cell function

Although the molecular mechanisms by which AHLs trigger host cell damage have not been identified and research on the targets for the identified receptors of AHL in mammalian cells is still at an early stage, several signaling pathways and mechanisms mediating the activity of AHL have been elucidated. Typically, the host activates the innate immune system through pattern recognition receptors (PPRs) when pathogenic microorganisms and their metabolites invade. As one type of PRR, Toll-like receptors (TLRs) are capable of directly activating an immune response.^80^ AHL has been reported to modulate neutrophil phagocytosis ^64,81^ and inhibit DC antigen presentation,^56^ thus inducing pro-inflammatory cytokines and exacerbating airway inflammation.^53,82^ AHL has also been reported to counteract lipopolysaccharide-induced nuclear factor-κB (NF-κB) activation,^83^ thus triggering lymphocyte death.^61^ It has been shown that TLRs are not necessary for the host response to AHL.^84^ In a previous study, we found that NF-κB phosphorylation did not change despite altered expression of TLRs after AHL treatment. Furthermore, inhibitors of NF-κB transcriptional activity did not alleviate AHL-induced oxidative stress and cell viability. These results indicate that the TLR–NF-κB signaling pathway is not involved in AHL-induced host cell damage.^85^ Thus, the effector functions of AHL appear to occur through a unique signaling platform.

**Figure 2.**
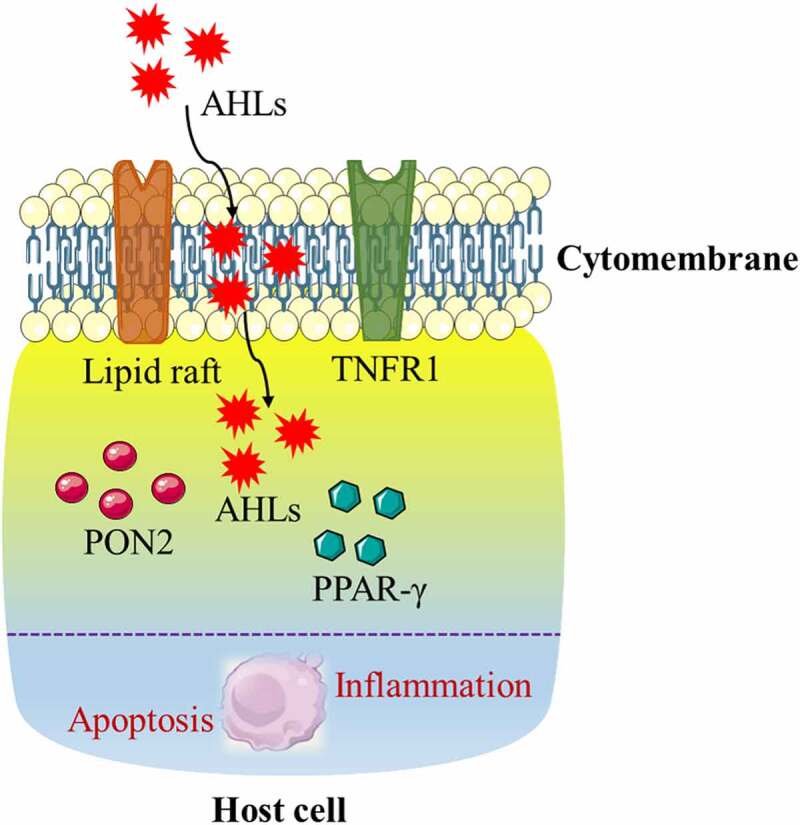
Proposed model of the potential mechanism of N-acyl-homoserine lactones (AHLs) mediating the biological effects in host cells. The lipid raft and tumor necrosis factor receptor (TNFR1) located on the cell membrane, as well as paraoxonase 2 (PON2) and peroxisome proliferator-activated receptor-γ (PPAR-γ) located in the cytoplasm, are potential receptors of AHLs, which may collectively induce apoptosis and the inflammatory response in host cells with AHLs.

The lipid raft is a micro-domain on the plasma membrane that is rich in cholesterol and sphingomyelin. The lipid raft is like a platform for protein parking that is closely related to membrane signal transduction and protein sorting. Lipid rafts may initially form in the endoplasmic reticulum, and some lipid rafts can be cross-linked with submembrane cytoskeletal proteins to varying degrees after being transported to the cell membrane.^86–89^ Presently, there is no consensus on whether lipid rafts mediate the harmful biological functions of AHLs on the host. Because AHL is a kind of lipid-soluble small molecule, it has been reported that cholesterol on the cell membrane is a potential receptor for AHL, and AHL can be transported into the cell through lipid rafts on the cell membrane to perform specific biological functions. The destroyer of lipid rafts, MβCD, disrupts the structure of lipid rafts by removing cholesterol from the cell membrane. It can effectively inhibit the increase in the permeability of Caco-2 cells induced by AHL.^90^ However, another report indicates that AHL can enter the host cell through a passive transport pathway and has little interaction with the cell membrane.^42^ The reason for this discrepancy in findings may be due to the different cell types that were used, but the relationship between lipid rafts and AHLs still needs further research (Figure 2).

Currently, the mechanism by which AHLs regulate host cell function is unclear, and the receptors for host cell acceptance of AHLs have not been identified.^91^ However, it has been proposed that peroxisome proliferator-activated receptor-γ (PPAR-γ) may serve as a potential receptor for mammalian AHL. PPAR-γ is a nuclear receptor that acts as a transcription factor to regulate lipid metabolism. Previous studies on PPAR-γ have focused on the regulation of adipocyte differentiation, whereas few studies have addressed its mediation of apoptosis. In vitro studies have also confirmed that PPAR-γ agonists can induce apoptosis in cultured T cells in vitro, suggesting that it is a potential anti-inflammatory molecule that plays a role in immune dysfunction during a severe inflammatory response.^92^ An increase in PPAR-γ expression can also induce apoptosis in cancer cells.^93^ However, the opposite is true for neuronal cells, where either PPAR-γ overexpression or its receptor agonist rosiglitazone can exert an anti-apoptotic effect by protecting mitochondria through the upregulation of anti-apoptotic proteins ^94^ (Figure 2).

Paraoxonase 2 (PON2) is widely expressed in mammalian tissues, and its main function is to hydrolyze QSSM AHL. Therefore, PON2 may be a potential regulator of AHL to exert its biological functions. Presently, there is no agreement on the role of PON2 in AHL in regulating host cell function. Studies have shown that PON2 has antioxidative, anti-inflammatory, and anti-apoptotic properties in human aortic endothelial cells, depending on its hydrolytic capacity.^95–97^ In support of this postulate, investigators have reported that PON2 can attenuate AHL-mediated biological effects that impair host cell function, based on its antioxidative and anti-apoptotic activities. By contrast, a recent study has shown that AHL induces apoptosis of mouse embryonic fibroblasts in a PON2-dependent manner.^98^ Subsequent studies have shown that PON2 hydrolyzes AHL to AHL-acids and stores them in mitochondria, which subsequently results in a series of biological effects in mammalian cells ^76,99^ (Figure 2).

During *Pseudomonas aeruginosa* infection, the signal molecule AHL is secreted and sensed by neighboring bacteria through the quorum sensing system. AHL binds to LasR, leading to its own syngeneic synthesis. In the model of AHL treatment of host immune cells, a portion of AHL is incorporated into the host cell membrane, leading to the destruction of lipid domains, that is, the unique ordered lipid domains in eukaryotic cell membranes that are soluble in the presence of AHL. This in turn leads to the forced expulsion of tumor necrosis factor receptor 1 (TNFR1) to the disordered phase of the membrane, leading to a higher degree of spontaneous trimerization and TNFR1 signaling in the absence of external ligands. This distribution change alone drives the entire process of caspase 8 and caspase 3-axis activation and apoptosis, and it is used by *Pseudomonas aeruginosa* to suppress host immunity. Therefore, *Pseudomonas aeruginosa* gains a survival advantage when the host defense system is weakened. In this case, the toxicity of AHL is at least partially achieved by actively hijacking the host cell death signaling pathway. These findings indicate a previously unknown mechanism of how eukaryotic cells sense microbial metabolites ^100^ (Figure 2).

## Effects and potential mechanisms of QSSM AHL on host intestinal health

### Impact of AHL on human and mouse intestinal health

The presence or absence of AHL in the mammalian gut is an unresolved issue in the scientific community. It has been suggested that the use of LuxR-based biosensors may not be able to detect AHL in the gut due to their detection limits being too high;^101,102^ thus, there is a need to develop new and more sensitive methods.^103^ Kumari et al. developed a whole cell sensor system for detecting AHLs and showed that these signaling molecules detected in saliva and feces may be potential endogenous biomarkers for gastrointestinal diseases.^104^ LC/MS-based methods were also used to detect AHLs in biological samples.^105–108^ Furthermore, recent studies have developed a UPLC-MS/MS-based assay method to screen for 27 AHLs in gut microbiota and hosts. Various AHL molecules were identified in fecal content, serum, and the liver of conventionally-raised mice, but not in germ-free mice. Pathogen-produced C4-HSL was detected in fecal content and serum of mice infected with *Citrobacter rodentium*, but not in uninfected mice. In addition, *Citrobacter rodentium* infection significantly increased the serum levels of several AHL molecules. This study suggested that gut microbiota can produce a variety of AHLs and transport them into the host.^109^ Furthermore, a team of investigators detected 14 different AHLs in the feces of IBD patients and healthy individuals by using mass spectrometry, and the distribution of AHLs correlated with disease status.^110^

### Impact of AHL on ruminant and pig intestinal health

In developed countries, such as Europe and the United States, cereal foods are widely used in the feeding of ruminants because they are economical. Due to the lack of quality forage, high percentage grain concentrate diets are also widely used in the feeding of ruminants in China to maximize the growth rate and economic efficiency of the animals. Administering high concentrate diets to dairy cows during specific physiological periods can effectively increase milk production.^111^ However, long-term feeding can lower the pH of the gastrointestinal tract and cause excessive accumulation of volatile fatty acids (VFAs), leading to subacute rumen acidosis (SARA).^112–117^ It has been reported that the prevalence of SARA in early and mid-lactation cows can reach 19% and 26%, respectively.^118,119^ SARA does not present serious clinical symptoms, but can cause physical damage to the mucosa of the digestive tract, and bacteria and bacterial products can easily invade the systemic circulation, which can then lead to diseases such as mastitis, laminitis, and hepatapostema, ultimately affecting ruminant health and performance and causing significant economic losses.^120,121^ Statistically, SARA costs the U.S. dairy industry $1 billion annually.^122^ Erickson et al. ^102^ first studied the secretion of the AHL signaling molecule in the digestive tract of ruminants. The authors detected AHL in the rumen fluid of cattle fed at different concentrations in 80% of the animals. Moreover, AHL in the rumen fluid of ruminants fed a high concentrate diet had longer side chains than those fed a low concentrate diet. These results indicate that AHL does exist in the digestive tract of ruminants, and high concentrate diets can affect the secretion of AHL in the digestive tract to a certain extent. Furthermore, Edrington et al. ^123^ found that AHL was not detected in hindgut content samples of ruminants in all seasons by studying the effects of seasons on the secretion of AHL. The authors explained this phenomenon from two aspects: i) Under normal physiological conditions, the pH in the posterior segment of the intestine is alkaline, which leads to the opening of the AHL and its inactivation; ii) There may be a lack of microbes in the gut that produce AHL. However, after feeding the animals with a high concentrate diet, excessive fermentation in the posterior segment of the intestines of ruminants caused hindgut acidosis, which greatly reduced the pH in the intestinal lumen. As such, this may provide favorable conditions for the stabilization of the AHL structure. In our study, it was found that after ruminants were fed a high concentrate diet, the intestinal acidity decreased and the microflora drastically changed, which may increase the number of pathogenic microorganisms and certain conditional pathogens, eventually leading to the secretion of AHL.^124–128^ So far, it is not clear whether AHL has any effect on the functional status of the host cells in the posterior segment of the gut of ruminants, let alone its potential mechanism.

The pig industry occupies a very important position in China<apos;>s economy. However, the pig<apos;>s birth weight and individual production performance variations in the breeding process have brought great inconvenience to the management of large-scale pig farms in terms of barn utilization, disease prevention, and feed preparation. Under modern breeding conditions, although pigs have the same genetic background and breeding environment, there are still obvious variations between their individual production performance.^129,130^ IUGR leads to the low birth weight of piglets, followed by high mortality during lactation and low growth performance throughout the life period. These are the outstanding problems in the pig industry.^131^ According to statistics, 15% to 20% of IUGR piglets in China have a birth weight less than 1.1 kg.^132^ IUGR piglets are usually accompanied by intestinal dysfunction after birth, which affects the animal<apos;>s production potential after birth.^133–135^ Compared with normal birth weight piglets, the feed utilization efficiency of IUGR piglets in the later stage is reduced by 30%, and the average time to slaughter is extended by 30 days. The annual economic loss to China<apos;>s pig production is as high as 15 billion. Furthermore, the impact of IUGR on the intestinal health of pigs can continue until the growth period.^136–140^ By systematically analyzing the function of the epithelial barrier in the hindgut (colon and cecum) of growing pigs, we found that IUGR leads to a persistent deterioration of the morphological structure, oxidative stress, and apoptosis. Furthermore, IUGR leads to impaired epithelial barrier function in the hindgut of growing pigs, and the proliferation of gram-negative bacteria in the intestine of pigs may play an important role.^141^ It has been shown that gram-negative bacteria regulate the function of eukaryotic cells by secreting AHL, which disrupts the homeostasis of host intestinal epithelial cells and ultimately leads to intestinal epithelial barrier dysfunction.^142^ It was shown that gram-negative bacteria, namely, *Bacteroides* and *Clostridium*, are the dominant microbiota in the intestine of IUGR pigs.^143^ The proliferation of gram-negative bacteria in the intestinal lumen of IUGR piglets suggests that the concentration of AHL may be significantly increased in the intestine of IUGR pigs, and the deterioration of intestinal epithelial barrier function in IUGR pigs may be triggered by AHL. We further examined nine AHLs, including 3-oxo-C12-HSL and its analogs, and found that the concentrations of two AHL (3-oxo-C12-HSL and 3-oxo-C14-HSL) were significantly higher in the feces of IUGR pigs than those of normal birth weight pigs. Spearman correlation analysis showed a strong correlation between 3-oxo-C12-HSL and different microorganisms in the feces of IUGR pigs. In vitro cellular assay studies revealed that 3-oxo-C12-HSL impaired IPEC-J2 cell viability in a dose-dependent manner. By further exploring the molecular mechanism of 3-oxo-C12-HSL-induced damage in intestinal epithelial cells, we found that 3-oxo-C12-HSL mainly altered the “import across plasma membrane” and “arginine and proline metabolism” pathways in intestinal epithelial cells.^144^

### Potential mechanisms by which AHL impairs intestinal health

**Figure 3.**
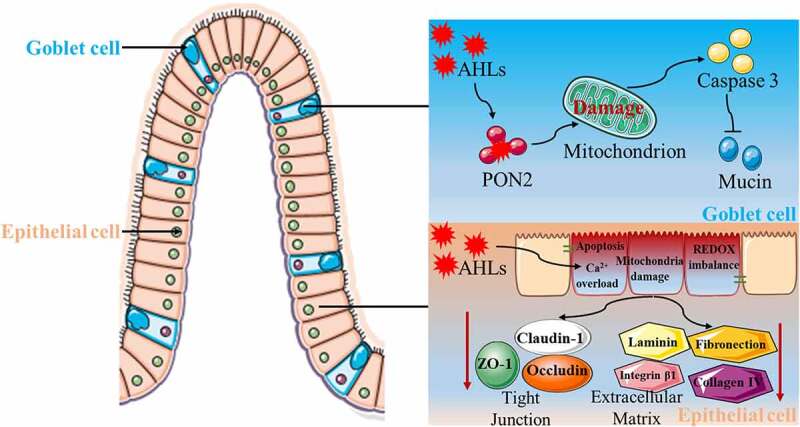
Proposed model of modulation of host gut damage by N-acyl-homoserine lactones (AHLs)-driven quorum sensing. Under the catalytic action of paraoxonase 2 (PON2), which activates the expression of caspase3, there is an induction of intestinal goblet cell apoptosis, an inhibition of mucin synthesis and secretion, and ultimately a destruction of the intestinal mucus barrier. AHLs induced calcium ion overload, mitochondrial damage, REDOX imbalance, and apoptosis of intestinal epithelial cells, but inhibited the expression of tight junctional and extracellular matrix proteins, thus destroying the intestinal epithelial cell barrier.

The gut<apos;>s first line of defense against invasion of pathogenic microorganisms and harmful metabolites is the mucus layer that covers the outer side of intestinal epithelial cells.^145,146^ The main component of the mucus layer is mucin, which is synthesized and secreted by intestinal goblet cells and acts as a barrier covering the entire intestinal surface.^147–149^ Studies have shown that the absence or abnormal expression of mucins can lead to intestinal diseases, whereas pathogenic microorganisms and some of their metabolites can induce abnormal expression of mucins.^150–152^ As for the influence of AHL on the intestinal mucus barrier, we first established a co-culture model of 3-oxo-C12-HSL and intestinal goblet cells, and demonstrated that 3-oxo-C12-HSL induced an imbalance of intestinal goblet cell homeostasis by causing mitochondrial swelling, mitochondrial membrane potential depolarization, mitochondrial dysfunction, and cell apoptosis. Furthermore, 3-oxo-C12-HSL inhibits mucin synthesis and sulfuration, ultimately destroying the intestinal mucus barrier. On this basis, the 3-oxo-C12-HSL/PON2 specific inhibitor/intestinal goblet cell co-culture model was established, and it was found that 3-oxo-C12-HSL induced a series of harmful biological effects in a PON2-dependent manner, eventually resulting in the disorder of intestinal goblet cell structure and function ^85,153,154^ (Figure 3).

The physical barrier is an important feature of the intestinal barrier system. Its main function is to ensure the absorption of essential nutrients and prevent the translocation of pathogenic substances. Therefore, the physical barrier needs to have the characteristic of selective penetration.^155^ Under normal circumstances, substances in the intestinal lumen cross the intestinal epithelial barrier through cell bypass and transcellular pathways. The completion of the cell bypass pathway requires the participation of junctional complexes located at the apical region of intestinal epithelial cells.^156,157^ The tight junctional complex between cells is mainly composed of tight junctions, adhesion junctions, and desmosomes. Tight junctions are a multi-protein complex composed of transmembrane proteins, peripheral membrane proteins, and regulatory molecules, which mainly include the claudin, occludin, and zona occludens (ZO) families.^158^ The degradation, phosphorylation, or redistribution of proteins in the tight junctional complex can lead to changes in tight junction structure and function, disruption of the cytoskeleton or decreased transmembrane resistance of epithelial cells, increased epithelial permeability, disruption of epithelial barrier integrity, and dysfunction of the barrier. Therefore, maintaining the normal expression and distribution of tight junctional proteins between intestinal epithelial cells is essential for the function of the intestinal epithelial barrier. In recent years, many studies have shown that 3-oxo-C12-HSL can cause intestinal barrier dysfunction by destroying tight junctions between epithelial cells. A study on the human colon Caco-2 cell line showed that 3-oxo-C12-HSL can impair barrier function by reducing the expression and intracellular localization of tight junctional proteins, and induce matrix metalloprotease vitality loss, thus destroying barrier permeability and disrupting barrier function.^90^ Vikstrom et al. found that 3-oxo-C12-HSL compromised the integrity of Caco-2 cells by decreasing transepithelial intercellular resistance, reducing the expression and distribution of ZO-1 and occluding, and redistributing F-actin proteins.^77,159^ Our research showed that 3-oxo-C12-HSL destroys the function of the intestinal epithelial cell barrier by inducing apoptosis, triggering mitochondrial dysfunction and redox imbalance, degrading the extracellular matrix, and disrupting tight junctions ^160^ (Figure 3).

## Conclusion

In summary, the knowledge of the microbial QS has been greatly improved in recent decades, and the interactive effects of the microbial QSSM with mammalian host cells have been studied in great depth. On this basis, we summarized the effects of AHL, a signaling molecule produced by gram-negative bacteria, on host cells and its potential mechanisms. Furthermore, we similarly reviewed the relationship between AHL and certain intestinal diseases and confirmed 3-oxo-C12-HSL as an AHL that plays a key role in the development of metabolic diseases such as SARA and IUGR, which may help to guide us in interventions of bacterial infections by intercepting QS signals.

Despite the rapid progress of studies related to AHL that affect the physiological status of mammalian cells, many questions remain to be addressed. i) Current studies on AHL-host interactions were largely limited to in vitro cell culture experiments, and there is an urgent need to understand whether AHL can exert deleterious biological effects by coordinating the behavior of microorganisms and modulating microbial networks or single bacteria through animal experiments. Recently, we used SPF and GF mice as animal models, which were combined with fecal microbial transplantation (FMT) technology, to discover that 3-oxo-C12-HSL causes intestinal barrier damage and systemic inflammation by perturbing the intestinal microbiome,^161^ but the specific mechanisms need to be further investigated. ii) The explicit source of intestinal luminal AHL is unclear. The scientific community eagerly awaits well-designed experiments with a large number of individuals, as well as advanced technologies (metagenomics, metabolomics, and culturomics) to trace the origin of AHL. iii) A combination of qualitative, quantitative, and localization methods is needed to analyze the trajectory of AHL movement between the intestinal lumen and intestinal epithelial cells to better study how AHL contacts intestinal epithelial cells and exerts its biological effects. In conclusion, there is still a long way to understand the co-evolutionary pattern of microbial QS and QSSMs in humans and animals, and to scientifically develop strategies to prevent and treat QS-dependent bacterial infectious diseases from a multi-channel perspective.

## Figures and Tables

**Table:** 

3-oxo-C12-HSL:	N-3-oxododecanoyl-homoserine lactone
AHL:	N-acyl-homoserine lactone
DC:	Dendritic cell; IUGR: Intrauterine growth restriction
MUC:	Mucin
NF-κB:	Nuclear factor-κB
QS:	Quorum sensing
QSSM:	Quorum sensing signaling molecule
PON2:	Paraoxonase 2
PPAR-γ:	Peroxisome proliferator-activated receptor-γ
PPR:	Pattern recognition receptor
PQS:	Pseusomonas qinolone signal; SARA: Subacute rumen acidosis
TLR:	Toll-like receptor
TNFR:	Tumor necrosis factor receptor
VFA:	Volatile fatty acid
